# Polyol pathway and modulation of ischemia-reperfusion injury in Type 2 diabetic BBZ rat hearts

**DOI:** 10.1186/1475-2840-7-33

**Published:** 2008-10-28

**Authors:** Qing Li, Yuying C Hwang, Radha Ananthakrishnan, Peter J Oates, Dennis Guberski, Ravichandran Ramasamy

**Affiliations:** 1 Division of Surgical Science, Department of Surgery, Columbia University College of Physicians and Surgeons, New York, NY 10032, USA; 2 Pfizer Global Research, Groton, CT, USA; 3 Biomedical Research Models Inc, Worcester, MA, USA

## Abstract

We investigated the role of polyol pathway enzymes aldose reductase (AR) and sorbitol dehydrogenase (SDH) in mediating injury due to ischemia-reperfusion (IR) in Type 2 diabetic BBZ rat hearts. Specifically, we investigated, (a) changes in glucose flux via cardiac AR and SDH as a function of diabetes duration, (b) ischemic injury and function after IR, (c) the effect of inhibition of AR or SDH on ischemic injury and function. Hearts isolated from BBZ rats, after 12 weeks or 48 weeks diabetes duration, and their non-diabetic littermates, were subjected to IR protocol. Myocardial function, substrate flux via AR and SDH, and tissue lactate:pyruvate (L/P) ratio (a measure of cytosolic NADH/NAD^+^), and lactate dehydrogenase (LDH) release (a marker of IR injury) were measured. Zopolrestat, and CP-470,711 were used to inhibit AR and SDH, respectively. Myocardial sorbitol and fructose content, and associated changes in L/P ratios were significantly higher in BBZ rats compared to non-diabetics, and increased with disease duration. Induction of IR resulted in increased ischemic injury, reduced ATP levels, increases in L/P ratio, and poor cardiac function in BBZ rat hearts, while inhibition of AR or SDH attenuated these changes and protected hearts from IR injury. These data indicate that AR and SDH are key modulators of myocardial IR injury in BBZ rat hearts and that inhibition of polyol pathway could in principle be used as a therapeutic adjunct for protection of ischemic myocardium in Type 2 diabetic patients.

## Introduction

Cardiovascular disease represents the major cause of morbidity and mortality in patients with diabetes. Diabetic patients with coronary artery disease have high morbidity and mortality due to cardiovascular complications, with the incidence of heart failure after myocardial infarction significantly greater in patients with diabetes than in non-diabetic patients [1-4]. Ventricular myocardial biopsies from diabetic patients exhibit significant increases in necrotic cardiomyocytes relative to biopsies from non-diabetic patients [5,6], indicating impairment in myocardial ischemic tolerance. In addition, diabetic patients also exhibit cardiomyopathy independent of coronary artery disease characterized by restrictive physiology [5-7]. Though clinical literature is incontrovertible in demonstrating increased cardiac injury in diabetes after myocardial infarction, experimental studies using animal models have yielded mixed results in this regard.

Studies investigating cardiovascular changes in Type 2 diabetic rats have employed spontaneously diabetic Otsuka Long-Evans Tokushima fatty (OLETF), Goto-Kakizaki (GK), and Zucker Diabetic Fatty (ZDF) rats [8-12]. A spontaneous Type 2 diabetic rat BBZDR/Wor (BBZ), from Biomedical Research Models (Worcester, MA) has been shown to closely mimic human disease. This model is characterized by spontaneous onset of diabetes at approximately 70 days of age, preceded by obesity. It shows insulin resistance with hyperglycemia and hyperinsulinemia as well as hyperlipidemia, hypercholestrolemia and mild hypertension [13-15].

The polyol pathway enzyme aldose reductase catalyzes the reduction of aldo sugars and other saturated and unsaturated aldehydes [16-20]. This enzyme constitutes the first and the rate limiting step of the polyol pathway. This pathway has been suggested to play an important role in the development of vascular and neurological complications in diabetes [21-26]. Inhibition of aldose reductase has been shown to ameliorate vascular and other complications in diabetics [21-26].

Our early studies using the Type 1 diabetic BB rat hearts demonstrated that aldose reductase is a key component of ischemia-reperfusion injury and that inhibition of this pathway reduced injury and improved functional and metabolic recovery on reperfusion [27,28]. In this study, we investigated the role of polyol pathway in mediating injury due to ischemia-reperfusion in Type 2 diabetic BBZ rat hearts. The results from Type 2 diabetic BBZ rat hearts in this study demonstrate (a) increased activities of polyol pathway enzymes aldose reductase and sorbitol dehydrogenase with duration of diabetes, (b) increased ischemic injury and poor functional recovery after ischemia-reperfusion, (c) reduced ischemic injury and improved functional recovery consequent to inhibition of aldose reductase or sorbitol dehydrogenase.

## Methods

All studies were performed with the approval of the Institutional Animal Care and Use Committee at Columbia University, New York. This investigation conforms with the *Guide for the Care and Use of Laboratory Animals *published by the US National Institutes of Health (NIH publication No. 85-23, 1996). In our studies we chose the Type 2 BBZDR/Wor diabetic rats. The Type 2 BBZDR/Wor rats are characterized by spontaneous onset of diabetes at about 70 days of age, which is preceded by obesity. These rats also exhibit insulin resistance with hyperglycemia and hyperinsulinemia, as well as hyperlipidemia, hypercholesterolemia, and mild hypertension [13-15]. The lean non-diabetic littermates were use as controls in this study. All rats were received from Biomedical Research Models, Worcester, MA.

### Isolated Perfused Heart Model

Rats (300–400 g) were anesthetized by injecting ketamine/xylazine (80 mg/kg and 10 mg/kg; I.P.) and the hearts rapidly excised and placed in ice cold saline. Hearts were retrograde perfused (in a non-recirculating mode) through the aorta using an isovolumic perfusion system as published earlier [29,30]. Left ventricular developed pressure (LVDP), and left ventricular end diastolic pressure (EDP) were measured using a fluid filled latex balloon in the left ventricle with high pressure tubing connected to a pressure transducer [29,30]. Cardiac function measurements were recorded on a 4-channel ADI recorder. The perfusate consisted of NaCl 118 mM, KCl 4.7 mM, CaCl_2 _1.2 mM, MgCl_2 _1.2 mM, NaHCO_3 _25 mM, glucose 5 mM, palmitate 0.4 mM, bovine serum albumin 0.4 mM, and insulin 70 milliunits/L. The perfusion apparatus was tightly temperature controlled, with heated baths being used for the perfusate and for the water jacketing around the perfusion tubing to maintain heart temperature at 37 ± 0.2°C under all conditions. The oxygenation chamber in our perfusion system maintains perfusate Po_2 _> 600 mmHg. All rat hearts were paced at 300 beats/min with the use of pacing electrodes placed on the right atrium.

Hearts were subjected to 30 min of normoxic perfusion at a flow rate of 12.5 ml/min followed by 50 min of severe *low-flow *ischemia (perfusate flow reduced to 0.7 ml/min) and 60 min of reperfusion at a normal flow rate (12.5 ml/min).

To determine the mechanisms by which flux via aldose reductase impacts ischemic injury, experiments were performed in the presence of inhibitors of AR (ARI) or sorbitol dehydrogenase (SDI). The studies involving the use of SDI were to establish if the flux via SDH and accompanying increases in cytosolic NADH/NAD^+ ^are important event that mediates ischemic injury. After the equilibration period of 30 min, hearts (n = 6) from Type 2 BBZ and non-diabetic littermate rats were perfused with modified Krebs-Henseleit buffer containing 1 μM ARI zopolrestat (unbound [31]) or 200 nM SDI CP-470,711 starting 10 min prior to ischemia and continued throughout ischemia and reperfusion. The dose of ARI and SDI used here were based on our earlier studies [32-35].

### Biochemical Assays

Tissue levels of lactate, pyruvate, sorbitol, and fructose were measured as published by us earlier [32-35]. Integrated release of lactate dehydrogenase during reperfusion, a marker of ischemic injury [36], was measured as previously published in the literature.

### Western blot studies

Extracts from rat hearts were prepared by tissue homogenization in cell lysis buffer as previously described [32-34] and were subjected to further analysis. The protein concentration was determined using a DC Protein Assay kit (Bio-Rad). Equal amounts of protein were separated by SDS-PAGE (4–12% gradient gels), and proteins were transferred to a polyvinylidene difluoride membrane (Invitrogen), that was probed with primary antibodies. The antibodies were diluted at 1:1,000. The primary antibodies used were anti-aldose reductase IgG (custom prepared by Cambridge Research Biochemicals, UK), anti-sorbitol dehydrogenase IgG (custom prepared by Cambridge Research Biochemicals, UK), and anti-β-actin IgG (Sigma). Blots were visualized with a Phototop-Horseradish Peroxidase Western Blot Detection System (Cell Signaling), and quantitative analysis was performed using Image Quant TL software (Amersham).

#### Statistical Methods

Data were analyzed using INSTAT (GraphPad, San Diego, CA) software operating on an IBM compatible personal computer. Differences between different groups were assessed using ANOVA with subsequent Student-Newman-Keuls multiple comparisons post-tests if the p value for ANOVA was significant. A p value of less than 0.05 was used to reject the null hypothesis. All data are expressed as mean ± SD.

## Results

### Animal Characteristics

Body weight, heart weight, and serum metabolite data for age-matched non-diabetic (ND) and Type 2 diabetic (DM) rats are summarized in Table 1. As expected, at 12 and 48 weeks of diabetes, DM rats were significantly heavier than their age-matched ND counterparts (p < 0.05). Serum glucose was elevated in the DM group; since the animals were in the fed state, this presumably reflects the glucose intolerance present at this age. In both the diabetic groups (12 weeks & 48 weeks duration), glucose levels were elevated by about 4 fold in DM vs ND rats (p < 0.05). Fatty acids were also elevated in both the 12 weeks and 48 weeks diabetic rats compared to their age-matched non-diabetics (p < 0.05). Insulin levels in 12-week DM rats were higher than in age matched ND rats. In the 48-week DM rats, insulin declined markedly and was significantly lower than in the age-matched ND rats. These data are entirely consistent with previously published data on Type 2 diabetic rats [37,38] and clearly demonstrate that the BBZ rats exhibit a Type 2 diabetic phenotype.

**Table 1:** Body weight and serum metabolite data from aged matched non-diabetic (ND) and Type 2 diabetic rats (DM)

	**12 wk**		**48 wk**	
	
	**ND (n = 9)**	**DM (n = 6)**	**ND (n = 7)**	**DM (n = 6)**
Body weight, g	292 ± 12	412 ± 29*	386 ± 32	472 ± 19*
Glucose, mM	8.1 ± 0.4	32.9 ± 4.2*	11.9 ± 1.1	46.6 ± 3.8*
FFA, mM	0.36 ± 0.3	0.51 ± 0.5*	0.65 ± 0.5	1.12 ± 0.09*
Insulin, nM	0.69 ± 0.09	2.59 ± 0.22*	0.51 ± 0.06	0.23 ± 0.05*^,#^

* P < 0.05 vs age matched ND rats, ^#^p < 0.05 vs 12 Wk DM rats.

### Polyol pathway in Type 2 diabetic rat hearts under baseline normoxic conditions

**Figure 1:**
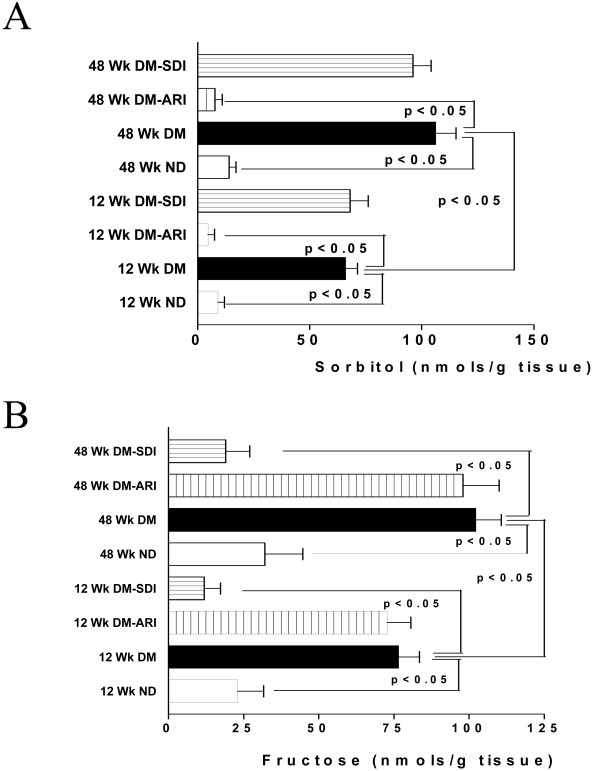
**Products of polyol pathway, sorbitol (A) and fructose (B) were measured in the hearts from 12 weeks and 48 weeks diabetic (DM) BBZ rats and their age matched non-diabetic (ND) controls.** Diabetes duration of 12 weeks (n = 9) and 48 weeks (n = 6) were used in this study. ARI denotes treatment with the aldose reductase inhibitor zopolrestat, while SDI denotes treatment with sorbitol dehydrogenase inhibitor.

To test the hypothesis that diabetes may increase flux via aldose reductase and sorbitol dehydrogenase, sorbitol and fructose content were measured in hearts under normoxic conditions from Type 2 diabetic rats (Fig 1A). Sorbitol and fructose content increased in Type 2 diabetic rat hearts compared to non-diabetic rat hearts (Fig 1A &1B). The changes in sorbitol and fructose were also observed as a function of diabetes duration. Inhibition of AR attenuated both sorbitol and fructose content in diabetic rats, whereas inhibition of SDH attenuated fructose content in diabetic hearts (Figure 1a &1b). These data are consistent with increases in flux via polyol pathway as a function of diabetes duration.

**Figure 2:**
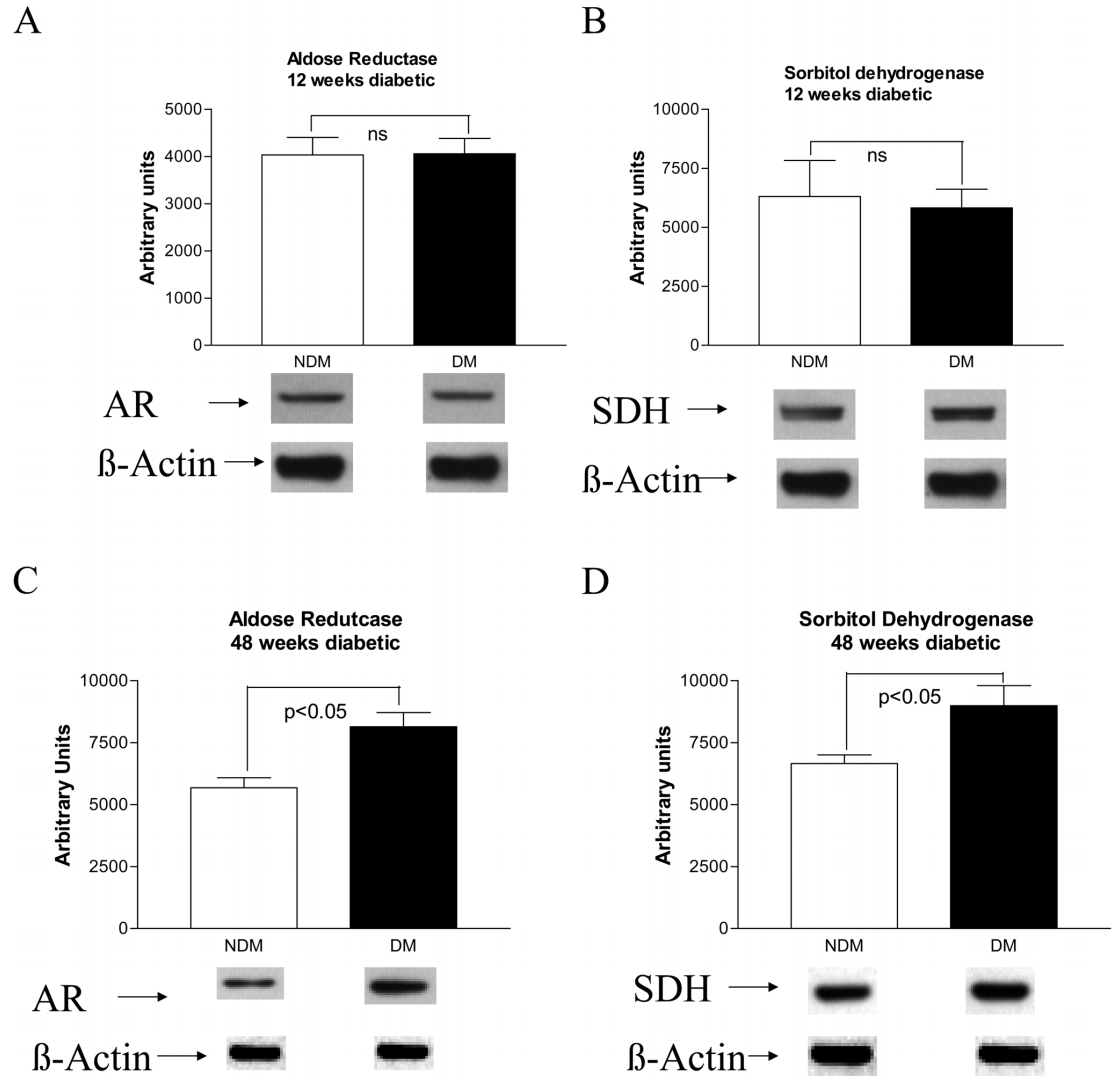
**Probing of the expression of aldose reductase (AR) (A) and sorbitol dehydrognease (SDH) (B) in hearts from 12 weeks diabetic BBZ and age matched non-diabetic littermate rat hearts using western blots.** Expression of AR (C) and SDH (D) were also probed in hearts from 48 weeks diabetic BBZ and age matched non-diabetic littermates. Equal amounts of protein were subjected to Western blotting using anti-AR IgG (A) or anti-SDH IgG; blots were then stripped and reprobed using anti-β actin IgG. Representative blots for each of the groups is presented here. Six hearts per group were used in this study.

Western blot analysis of AR and SDH protein expression in non-diabetic and 12 week diabetic hearts (Fig 2A–B) did not reveal any differences under baseline normoxic conditions, where as significant increases in AR and SDH expression levels were observed in 48 weeks diabetic hearts (Figure 2C &2D).

### Polyol pathway and ischemia-reperfusion injury in Type 2 diabetic rat hearts

**Figure 3:**
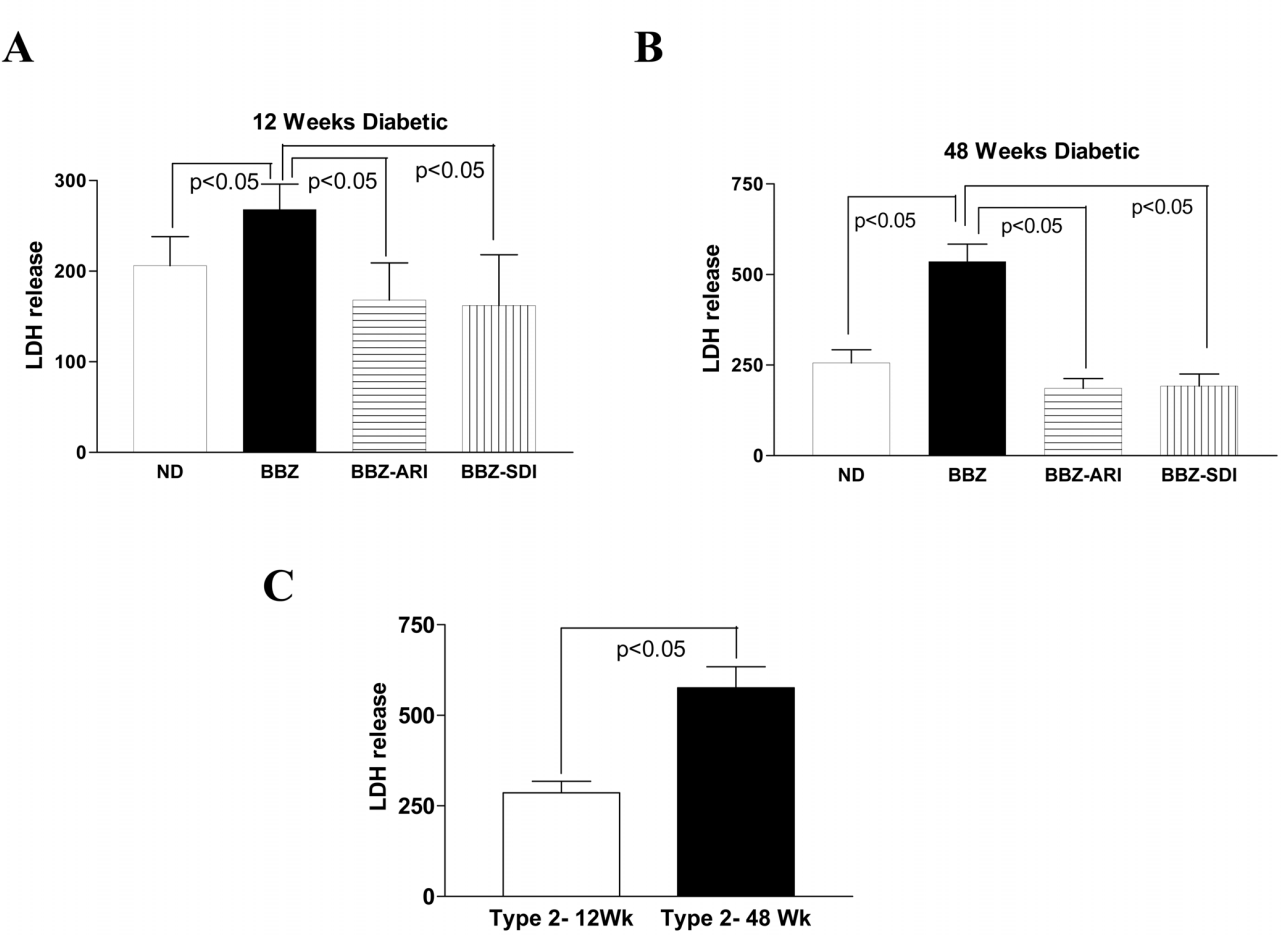
**Lactate dehydrogenase release (U/g dry wt) during 60 minutes of reperfusion, a marker of ischemic injury in age-matched non-diabetic (ND), Type 2 diabetic (BBZ), BBZ treated with aldose reductase inhibitor (BBZ-ARI), BBZ treated with sorbitol dehydrogenase inhibitor (BBZ-SDI) rat hearts.** Diabetes duration of 12 weeks (n = 9) (A) and 48 weeks (n = 6) (B) were used in this study. Comparison of LDH release in 12 weeks diabetic and 48 weeks diabetic BBZ rat hearts (C).

**Figure 4:**
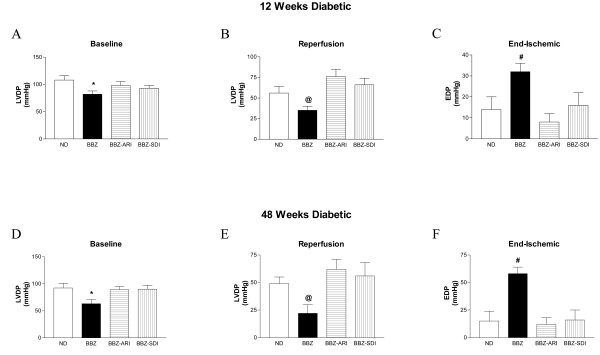
**Left ventricular developed pressure (LVDP) recovery, expressed as mmHg, in age matched non-diabetic (ND), Type 2 diabetic (BBZ), BBZ treated with aldose reductase inhibitor (BBZ-ARI), BBZ treated with sorbitol dehydrogenase inhibitor (BBZ-SDI) rat hearts under baseline (A, D) and reperfusion (B, E) conditions.** Left ventricular end diastolic pressure (EDP), expressed as mmHg, in ND, BBZ, BBZ-ARI, BBZ-SDI at the end of ischemic period (C, F). Diabetes duration of 12 weeks (A, B, C) (n = 9) and 48 weeks (D, E, F) (n = 6) were used in this study. * P < 0.05 vs ND, BBZ-ARI, and BBZ-SDI within respective diabetes duration matched group comparing LVDP at baseline, ^@ ^P < 0.05 vs ND, BBZ-ARI, and BBZ-SDI within respective diabetes duration matched group comparing LVDP at reperfusion, ^#^P < 0.05 vs ND, BBZ-ARI, and BBZ-SDI within respective diabetes duration matched group comparing End-ischemic EDP.

LDH release on reperfusion, a marker of myocardial ischemia-reperfusion injury, was significantly greater in 12 week diabetic BBZ when compared on age matched non-diabetic controls (Figure 3A). Similarly, LDH release on reperfusion was also significantly greater in 48 weeks diabetic compared to its age matched non-diabetic rat hearts (Figure 3B). Comparison of LDH release indicated that injury after ischemia-reperfusion was significantly greater in 48 weeks diabetic than in 12-week diabetic rat hearts (Figure 4C). Treatment of diabetic hearts (12 weeks & 48 weeks) with an ARI or SDI attenuated LDH release, indicating protection of from ischemia-reperfusion injury (Figure 3A &3B). We have shown earlier that treatment with ARI or SDI protects non-diabetic rat hearts from ischemia-reperfusion injury [32-34].

### Polyol pathway and myocardial function during ischemia-reperfusion

**Figure 5:**
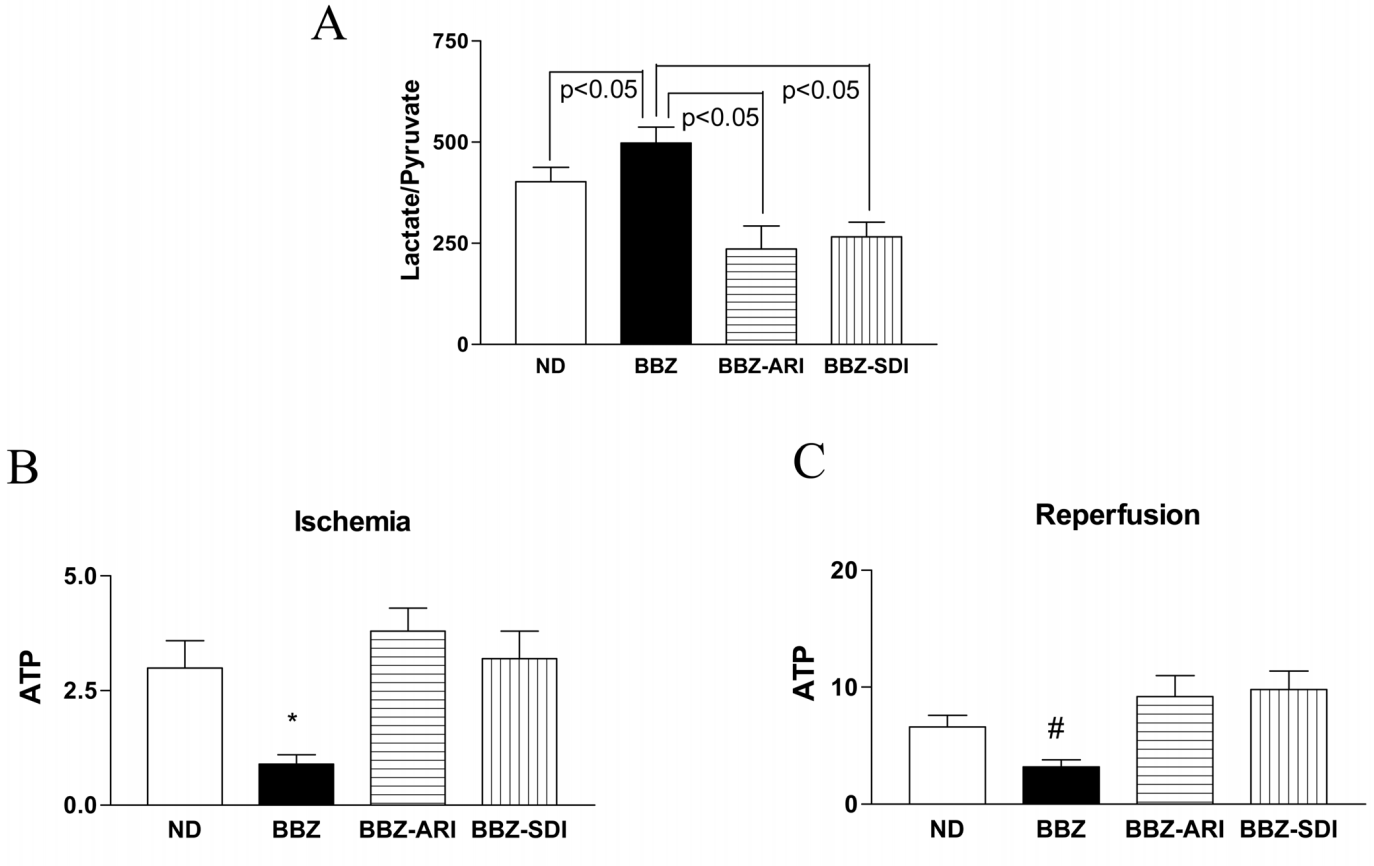
**(A) Lactate/pyruvate ratios in age matched non-diabetic (ND), Type 2 diabetic (BBZ), BBZ treated with aldose reductase inhibitor (BBZ-ARI), BBZ treated with sorbitol dehydrogenase inhibitor (BBZ-SDI) rat hearts at the end of ischemic period.** Six hearts were studied per group. ATP, expressed as μmoles/g dry wt, age matched non-diabetic (ND), Type 2 diabetic (BBZ), BBZ treated with aldose reductase inhibitor (BBZ-ARI), BBZ treated with sorbitol dehydrogenase inhibitor (BBZ-SDI) rat hearts at the end of ischemia (B), and at the end of reperfusion (C). Six hearts were studied per group. *P < 0.05 vs ND, BBZ-ARI, and BBZ-SDI comparing ATP at the end of Ischemia, ^#^P < 0.05 vs ND, BBZ-ARI, and BBZ-SDI comparing ATP at the end of reperfusion.

Figure 4 illustrates the changes in left ventricular developed pressure (LVDP), left ventricular end-diastolic pressure (EDP), and myocardial oxygen consumption in all four groups of hearts. LVEDP was set to 5–8 mmHg at the beginning of the perfusion period and increased in both the groups to a maximum after ~15 min of ischemia. LVDP under baseline conditions were significantly reduced in untreated 12-week diabetic compared to non-diabetic rat hearts (Figure 4A). After subjecting hearts to ischemia-reperfusion, LVDP recovery was significantly impaired in 12 week diabetic compared to non-diabetic control hearts (Figure 5B). Treatment of 12 weeks diabetic rat hearts with an ARI or SDI improved LVDP function under baseline (Figure 5A) and reperfusion conditions (Fig 4B). EDP at the end of ischemic period was significantly increased in untreated 12 week diabetic hearts (Figure 4C) compared to all other groups.

LVDP changes in untreated 48 weeks diabetic rat hearts were also impaired under baseline and reperfusion (Figure 4D, E respectively) in comparison with non-diabetic rat hearts. Treatment of the diabetic rat hearts with an ARI or SDI improved LVDP function under baseline and reperfusion conditions. End-ischemic EDP was significantly increased in 48 weeks diabetic rat hearts (Figure 4F) in comparison with non-diabetics. ARI and SDI treatment attenuated the increases in end-ischemic EDP in diabetic rat hearts. End ischemic EDP rose to 30 mm Hg in 12 week diabetic hearts, whereas it rose to 50 mmHg in 48 weeks diabetic hearts. The EDP data indicate that ischemic contracture was significantly greater with diabetes duration.

### Flux via Polyol pathway and lactate/pyruvate ratio

The lactate/pyruvate (L/P) ratio reflects the cytosolic ratio of NADH/NAD^+^. L/P ratios were measured in freeze-clamped heart tissue extracts. As shown in Table 2, the baseline L/P ratios were significantly higher in Type 2 diabetic hearts. Duration of diabetes further increased the L/P ratios. Pharmacological inhibition of aldose reductase or sorbitol dehydrogenase lowered the L/P ratio in Type 2 diabetic rat.

**Table 2:** Lactate:pyruvate ratios in Type 2 diabetic hearts

**Group**	**n**	**Lactate/Pyruvate ratio**
**Non-diabetic controls**	12	16.8 ± 3.2

**Type 2 diabetics**		
12 weeks duration	9	41.4 ± 8.8*
48 weeks duration	5	77.5 ± 12.2*

**Aldose reductase inhibited Type 2 diabetics**		
12 weeks duration	7	7.2 ± 1.8
48 weeks duration	5	10.5 ± 3.2

**Sorbitol dehydrogenase inhibited Type 2 diabetics**		
12 weeks duration	7	8.9 ± 2.1
48 weeks duration	5	12.6 ± 3.4

* p < 0.05 vs non-diabetic controls. The lactate/pyruvate ratios were measured after perfusion for one hour under normoxic conditions.

To determine if the rise in cytosolic NADH/NAD^+ ^ratio is a critical component of aldose reductase mediated ischemic injury, hearts from Type 2 were subjected to ischemia-reperfusion in the presence of AR or SDH inhibitor. Flux via aldose reductase and sorbitol dehydrogenase results in conversion of NAD^+ ^to NADH thus influencing cytosolic NADH/NAD^+ ^ratio; hence inhibition of either aldose reductase or sorbitol dehydrogenase would limit such changes. Figure 5A demonstrates that end ischemic cytosolic L/P ratio was reduced in Type 2 diabetic rat hearts by inhibiting aldose reductase or sorbitol dehydrogenase. These data demonstrate that NADH/NAD^+ ^changes are linked to polyol pathway mediated ischemic injury in hearts.

### Polyol pathway and ATP changes during ischemia and reperfusion

To determine changes in myocardial energy metabolism, ATP content during ischemia and reperfusion was measured in all groups of hearts. ATP content was significantly reduced in untreated diabetic hearts compared to non-diabetics at the end of ischemia and reperfusion (Figure 5B &5C). Treatment with an ARI or SDI improved ATP content in diabetic rat hearts under all perfusion conditions (Figure 5B &5C).

## Discussion

Aldose reductase pathway has been implicated in the pathogenesis of diabetic vascular complications. In this study, we investigated if polyol pathway is an important player in mediating ischemia and reperfusion injury in type 2 diabetic BBZ rat hearts. The data presented here demonstrates that flux via aldose reductase and sorbitol dehydrogenase is increased in type 2 diabetic rat hearts and that these increases play key roles in determining the extent of ischemic damage.

Studies in BBZ rats showed significant increases in nerve sorbitol and fructose contents when compared to those in non-diabetic littermates [14]. It was also shown that increases in nerve sorbitol continued with duration of diabetes, whereas nerve fructose content did not follow that trend in the BBZ rat [14]. We show here that flux via aldose reductase and sorbitol dehydrogenase continues to increase with duration of diabetes in the BBZ rat hearts. Furthermore, the increases in protein expression of aldose reductase and sorbitol dehydrogenase coupled with increased hyperglycemia presented here may, in part, be responsible for the increased flux via these enzymes in 48 weeks diabetic BBZ rat hearts. However, it is well established that glucose flux through aldose reductase is increased in diabetes. Since aldose reductase has a high Km for glucose, it would mean that increasing hyperglycemia is likely to increase flux via polyol pathway proportionately. At 48 weeks of diabetes, the degree of hyperglycemia is significantly greater than at 12 weeks, with far lower insulin levels. The greater glucose availability over time (amount and duration) is, in part, a key reason for the increased flux through aldose reductase.

Flux via polyol pathway has been associated with changes in cytosolic NADH/NAD+ the ratio [32-35,39-41]. These changes in cytosolic redox ratio have been demonstrated in ischemic and diabetic state [32-35,39-41]. In this study, we demonstrate that cytosolic lactate/pyruvate ratio, a measure of cytosolic NADH/NAD, is associated with increases in polyol pathway activity in type 2 diabetic BBZ rat hearts. These data are consistent with our earlier studies [32-35], demonstrating increased flux via polyol pathway during ischemia resulting in increased cytosolic NADH/NAD+. The data presented here show that inhibition of aldose reductase or sorbitol dehydrogenase attenuates increases in cytosolic NADH/NAD ratio in type 2 diabetic rat hearts and is associated with changes in ATP levels and protection of hearts from IR injury.

Although the present results show that elevated NADH/NAD+ can be blocked by either ARI or SDI treatment in this preclinical model, extreme caution should be exercised in regard to extrapolation of SDH inhibition to the clinical arena. This is warranted based on a) reports of increased neuronal axonal dystrophy observed in rat ganglia, [42]. and b) the discontinuation of an SDI clinical trial for adverse events that possibly might have been mechanism-related (Landau Z et al., manuscript in submission). In the diabetic rat model, increased occurrence of ganglionic lesions with SDI treatment notably was prevented by concurrent administration of an ARI [43].

Aldose reductase pathway inhibition attenuated the rise in EDP during ischemia in diabetic hearts. Several studies have shown that the attenuation of the rise in EDP by interventions is linked to the maintenance of intracellular sodium and calcium homeostasis [44-49]. In our earlier studies, we have shown that inhibition of aldose reductase attenuated the rise in intracellular sodium and calcium during ischemia and reperfusion in Type 1 BB/WOR diabetic hearts [48]. Protection of the Type 2 diabetic ischemic hearts by aldose reductase or sorbitol dehydrogenase inhibitors in this study is presumably due to attenuation of changes in sodium and calcium homeostasis during ischemia – reperfusion. However, further studies are required to confirm this speculation.

Overwhelming clinical evidence indicate that the diabetic heart in humans is sensitive to ischemic injury [1-6]. However, the animal studies addressing this issue have resulted in ambiguous results. In the type 2 ZDF rat hearts, in-vivo ischemia – reperfusion resulted in increased infarct size and poor functional outcome [50]. A study by Wang & Chatham, using isolated perfused heart models, showed that despite greater contracture during low flow ischemia the diabetic ZDF rat hearts exhibited improved functional recovery on reperfusion [9]. Our studies presented here in isolated perfused hearts demonstrate that ischemia/reperfusion increases injury and impairs functional recovery in type 2 diabetic BBZ rat hearts. In our studies and in the one by Wang and Chatham [9], ischemic contracture during low flow ischemia was significantly greater in Type 2 diabetic rat hearts than in nondiabetics. In our studies, increased ischemic contracture was also associated with greater ischemic injury and poor functional recovery on reperfusion. Thus, the data presented in our study are consistent with earlier studies demonstrating association between ischemic contracture, ischemic injury and functional recovery.

Earlier studies by us [27,28,32,33,35] and others [51,52] demonstrated that inhibition of aldose reductase affords protection to myocardium against ischemia-reperfusion injury. It should be noted that injury to myocardium occurs both at the ischemic and reperfusion period. The presence of aldose reductase inhibitor throughout ischemic and reperfusion period indicates that injury during both ischemic and reperfusion phase was attenuated by aldose reductase inhibition.

One aspect of injury to the myocardium not addressed by our data is the role of highly reactive oxygen species in mediating injury in the Type 2 diabetic hearts. Excess generation of highly reactive free radicals, both due to hyperglycemia and ischemia-reperfusion injury [53] can exacerbate injury in diabetics. The impact of aldose reductase inhibition in attenuating highly reactive free radicals, during ischemia-reperfusion, needs to be examined in the Type 2 diabetic hearts.

## Conclusion

In summary, we demonstrate that fluxes via polyol pathway enzymes aldose reductase and sorbitol dehydrogenase are increased in type 2 diabetic BBZ rat hearts as a function of disease duration. The increases in flux via these enzymes were accompanied by increased lactate/pyruvate ratio. Inhibition of aldose reductase or sorbitol dehydrogenase reduced ischemic injury, improved functional recovery and ATP levels after ischemia – reperfusion in diabetic rat hearts. These data suggest that inhibition of polyol pathway could in principle be used as a therapeutic adjunct for protecting ischemic myocardium in type 2 diabetic patients.

## Abbreviations

AR: Aldose reductase; ARI: aldose reductase inhibitor; L/P: lactate/pyruvate; SDH: sorbitol dehydrogenase; SDI: sorbitol dehydrogenase inhibitor.

## Competing interests

PJO is an employee of the Pfizer Inc. and DG is the President of Biomedical Research Models. All the other authors have no competing interests.

## Authors' contributions

QL performed isolated perfused heart studies and participated in analysis of the data. YCH carried out the initial ischemia-reperfusion protocol and assisted in the design of the studies. RA carried out the biochemical and molecular studies and assisted in the analysis of data. PJO provided the reagents and participated in the manuscript preparation, DG provided the Type 2 diabetic rats and guided in the long term care of these animlas them. He also assisted in the intil study design. RR conceived of the study, participated in its design, coordination of data collection, and prepared the manuscript. All the authors read and approved the final manuscript.
